# Influence of two kinds of clearance joints on the dynamics of planar mechanical system based on a modified contact force model

**DOI:** 10.1038/s41598-023-47315-1

**Published:** 2023-11-23

**Authors:** Haiyan Tan, Li Li, Qiang Huang, Zhuoda Jiang, Qingxiang Li, Youming Zhang, Donglin Yu

**Affiliations:** 1 School of Mechanical & Intelligent Manufacturing, Jiujiang University, Jiujiang, 332005 China; 2 State Key Laboratory of Digital Manufacturing Equipment and Technology, School of Mechanical Science and Engineering, Huazhong University of Science and Technology, Wuhan, 430074 China; 3 South-Central Minzu University, Wuhan, 430074 China

**Keywords:** Mechanical engineering, Computational science

## Abstract

This study takes the slider-crank mechanism with revolute joint and translational joint as the research object and studies the contact force model of the clearance joint and the influence of the hybrid clearance joints on the nonlinear dynamic behavior of the mechanism. A modified contact force model is established based on the simplified elastic oscillator model, which can be used as a normal force in clearance joint. In the new contact force model, the component n of the indentation depth can be arbitrarily selected and it can support the calculation of contact force for both fully elastic recovery, non-elastic recovery and fully inelastic recovery. Based on the LuGre friction model, the tangential friction model of the clearance joint is given. Thus, the normal force and tangential force during the dynamic contact of the clearance joint are formed. Combining Lagrange’s equations of the first kind with the modified normal force and tangential friction force, the dynamic equations of the multi-body system with clearance joints are established. The Baumgarte stabilization method is used to improve the numerical stability. The correctness of the dynamic prediction model in the mechanism with clearance joint is verified by experiment. The dynamic analysis of the slider-crank mechanism with mixed clearance joints shows that the revolute clearance joint has a greater influence on the mechanism than the translational clearance, and the revolute clearance joint plays a leading role in the dynamic response.

## Introduction

Clearance is an objective existence between two moving parts in multibody systems, which will cause the internal collision of the connected components. The reaction force of the clearance joint will show a large continuous fluctuation phenomenon, which will seriously affect the dynamic response and reduce the dynamics performance of the mechanical system^1–3^. To analyze the influence of the clearance joint on the dynamic characteristics of the mechanism, the contact force model between the bearing and the journal should be established. The key problem is to establish a suitable contact force model during the collision^4,5^. Many researchers have studied the dynamics of contact in multi-body system^6,7^. The analysis methods in this field mainly include the continuous contact force method and the discontinuous contact force method^8,9^.The continuous method usually uses a continuous contact force model to represents the force generated by the collision and assumes that the force and deformation are continuously changing. The contact force model^10^ is described as a spring damping element, which can be divided into linear and nonlinear, the Kelvin-Voigt model belongs to the former, the latter such as the Hunt and Crossley mode^11^, the Lankarani and Nikravesh models^12^, which are on Hertz contact theory and damping effect. These models can describe the energy loss during the collision, the damping effect includes the coefficient of restitution of the collision. It should be pointed out that this model is established under the condition that the coefficient of restitution is close to 1. Flores^13^ derived a new contact force model that is not limited by the coefficient of restitution, but the exponent of the indentation depth was 1.5. The continuous analysis method considers that the interaction force between the collision bodies is continuous during the entire contact and collision process, considering the collision process, this method is more in line with the actual collision behavior, this method was applied in the following literature^1,9,14–16^. The discontinuous method is also called the impulse momentum method, it assumes that the contact collision is instantaneous, and the collision process is divided into two stages, namely, before and after collision. This method cannot determine the magnitude of the collision force during the collision, which is a relatively effective analysis method^17^. Scholars such as Khulief^18^ and Yigit^19^ studied the collision problem of multi-body systems based on the impulse-momentum method. Hong Jiazhen et al.^5^ applied this method to the research on the contact collision dynamics of the spacecraft extension mechanism. Rhee^20^ used this method to establish a dynamic model of the slider-crank mechanism considering clearance and friction. However, this method cannot determine the magnitude and action process of the collision force during the collision.

In recent years, the severe consequences of clearance joints on the dynamic response of mechanical systems have attracted many theoretical and experimental studies. However, these studies mainly focused on a planar system with a clearance joint. Flores^21^ studied the dynamic response of a multi-body system with multiple clearance hinges, with different parameters (such as clearance size, crank speed, and the number of clearance joints) on the dynamic performance of this type of system. Erkaya and Uzmay^2^ theoretically and experimentally studied the influence of the clearance in the slider-crank mechanism with two clearance joints on the vibration and noise characteristics of the mechanism. The researchers modeled the clearance in the joint as a massless rod whose length is the clearance size. However, the dynamic interaction of multiple clearance joints has not been studied. Tan^22,23^ used the continuous contact force method to study the coupling phenomenon between the two clearance revolute joints, and analyzed the influence of friction on mechanism dynamics. Bai^24,25^ and Muvengei^26–28^ studied the effect of clearance joints on the dynamic behavior of multi-body mechanical systems, mainly analyzing the dynamic behavior of several periods. Flores^21^ and Chen^29^ studied the nonlinear characteristics of multi-body mechanical systems with clearance joints through phase diagrams and Poincaré diagrams for different clearance sizes and crank speeds. Wang^30^ proposed a dynamic model considering different clearance sizes, crank speeds, and different materials in the multi-body system and verified the correctness of the simulation model with experiments. Tian^31^ provided a comprehensive overview of the analytical, numerical, and experimental approaches for the kinematic and dynamic analyses of multi-body mechanical systems with clearance joints based on five hundred references. Zhang and Wang^32^ investigated the dynamics of the mechanism with flexible slider and clearance translational joint, the numerical results showed that the small deformation of the slider and the size of clearance affect the dynamic response. Dupac and Beale^33^ studied the effect of translational clearance joint and linkage crack in mechanism, the results pointed out that the crack in linkage and the clearance at slider joint change the dynamic behavior of the multisystem. Flores^34^ used a non-smooth dynamic approach to model a planar rigid body system with translational clearance joint. The results show that the existence of clearance joints in a multibody system influences their dynamics response. Wu and Sun^35^ conducted numerical research on a double crank slider mechanism with translational clearance joint, and performed experimental verification.

Salahshoor^36^ investigated the effect of joint stiffness on the vibrational behavior of a typical crank-slider mechanism with flexible components and joint clearance. Li investigated the thermally induced vibration of solar arrays^37^ and the dynamics of the mechanism considering solid lubrication^14^. Li^38^ concerned with the effect of joint clearances in solar array systems, and the results indicated that joint clearances affect the dynamic behavior of the deployable mast of the solar array system. Shi^8^ presented a framework of a virtual prototyping environment for the design and analysis of the steam turbine reheat-stop-valve mechanism with clearance, developed a simulation strategy that integrated thermal behavior, valve mechanism dynamics and other factors. Recently, the research on the lubrication of clearance joints in multi body mechanisms has attracted more and more scholars’ attention, and a large number of research results have been achieved^39–43^. Ivo Roupa et al.^44^ studied multibody formulation with Fully Cartesian coordinates (FCC) for planar systems, and described the most relevant features of FCC.

Recently, some scholars have studied the dynamic effects of two types of clearance joints on multibody mechanisms. Wu^45^ used correlation dimension and bifurcation analysis to analyze the multi-clearance planar crank-slider mechanism. Xiao^46^ study the nonlinear dynamics of rigid-flexible coupling multi-link mechanism considering revolute clearance and translational clearance. In general, the research on the dynamic behavior of mechanisms with clearance joints mainly focuses on the dynamic behavior of multibody mechanisms with revolute or translational clearance joints and mechanisms with mixed clearance joints, there are few studies on the interaction and comparison of the two types of clearance joints. The main innovation of this paper is to propose an improved contact force model that is not limited by the size of the coefficient of restitution, based on this improved contact force model, a dynamic model of a four-bar mechanism with clearance joints is established, an experimental system of the mechanism with clearance articulation is established, and the experimental results verify the correctness of the dynamic model. The influence of different numbers and different types of clearance joints on the nonlinear dynamics of the mechanism is analyzed, meanwhile, the influence of the two types of clearance joints on the mechanism is compared. This work is organized as follows: In Sect. “Modeling joint with clearance”, we describe the clearance model of the revolute joint and translational joint. Section “Contact force model” analyzes the contact force model in the joints and proposes a modified normal contact force model. We establish the equations of motion for a multibody system with clearance joints in Sect. “Dynamic modeling of mechanism with clearance joint”. Section “Experimental test rig” performs experiment verification. The simulation calculation in Sect. “Simulation and results” reveals the influence of different numbers and types of clearance joints on the dynamic response of the mechanism.

## Modeling joint with clearance

### Modeling revolute joint with clearance

**Figure 1:**
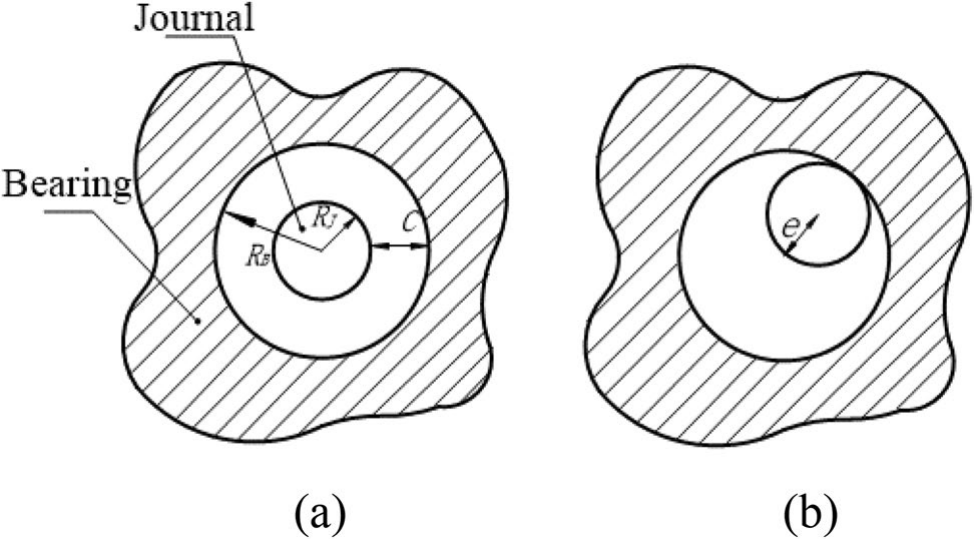
Clearance joint model.

Normally, the joint is ideal, the centers of journal and bearing are always coincident. After the clearance is introduced into the joint, their centers no longer coincide. The radius difference between the bearing and journal defines the radial clearance C, as shown in Fig. 1a. The eccentric vector **e** describes the relative motion relationship between the journal and bearing, as shown in Fig. 1b.

The radial clearance c, is described as,

1 $$c = R_{B} - R_{J} ,$$

where $R_{B}$ and $R_{J}$ are, respectively, the radii of the bearing and journal.

**Figure 2:**
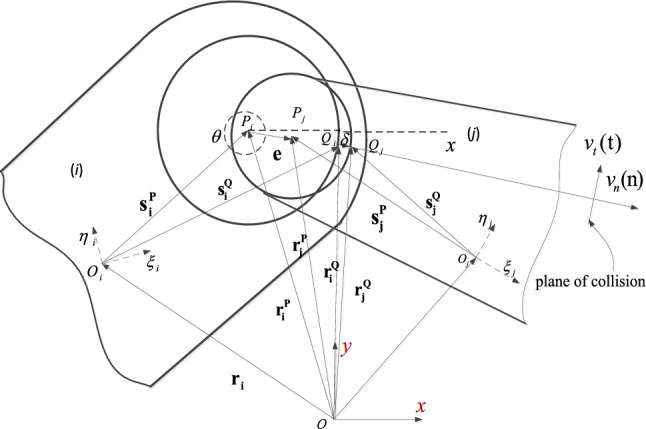
Revolute joint model with clearance in a multibody mechanical system.

In Fig. 2, $P_{i}$ and $P_{j}$ are the center of the bearing and journal respectively, ${\mathbf{e}}$ is eccentric vector connecting $P_{i}$ and $P_{j}$. is the derivative of the vector ${\mathbf{e}}$ concerning time.

2 $$\begin{gathered} {\mathbf{e}}{ = }{\mathbf{r}}_{j}^{P} - {\mathbf{r}}_{i}^{P} \hfill \\ {\dot{\mathbf{e}}} = {\dot{\mathbf{r}}}_{j}^{P} - {\dot{\mathbf{r}}}_{i}^{P} , \hfill \\ \end{gathered}$$

where ${\mathbf{r}}_{i}^{P}$ and ${\mathbf{r}}_{j}^{P}$ are described in the global coordinates reference frame, ${\dot{\text{r}}}_{k}^{P} (k = i,j)$ are the derivatives of ${\mathbf{r}}_{k}^{P}$ with respect to time.

3 $$\begin{gathered} {\mathbf{r}}_{k}^{P} = {\mathbf{r}}_{k} + {\mathbf{s}}_{k}^{p} {\kern 1pt} {\kern 1pt} {\kern 1pt} = {\kern 1pt} {\kern 1pt} {\kern 1pt} {\kern 1pt} {\mathbf{r}}_{k} + {\mathbf{A}}_{k} s{\prime}_{k}^{p} {\kern 1pt} {\kern 1pt} {\kern 1pt} {\kern 1pt} {\kern 1pt} {\kern 1pt} {\kern 1pt} (k = i,j) \hfill \\ {\dot{\mathbf{r}}}_{k}^{P} = {\dot{\mathbf{r}}}_{k} + {\dot{\mathbf{A}}}_{k} s{\prime}_{k}^{p} . \hfill \\ \end{gathered}$$

Here,${\mathbf{A}}_{k} (k = i,j)$ is the rotational transformation matrix for the body $k$, $${\mathbf{A}}_{k} = \left[ {\begin{array}{*{20}c} {\cos \phi_{k} } & { - \sin \phi_{k} } \\ {\sin \phi_{k} } & {\cos \phi_{k} } \\ \end{array} } \right]$$, $(k = i,j)$, $\phi_{k}$ is the angular displacement of the local coordinate system of the component $k$.

The magnitude of the eccentric vector is $e = \sqrt {{\mathbf{e}}^{{\mathbf{T}}} {\mathbf{e}}}$, ${\mathbf{e}}^{{\mathbf{T}}}$ is the transpose of ${\mathbf{e}}$, its unit vector can be expressed as,

4 $${\mathbf{n}}{ = }{\mathbf{e}}{/}e.$$

The direction of the unit vector ${\mathbf{n}}$ is the same as the centerline of the bearing and the journal, as shown in Fig. 2.

The penetration depth between journal and bearing is calculated as follows,

5 $$\delta { = }e - c.$$

$e$ is the size of the eccentric vector, $c$ is the radial clearance size.

$Q_{i}$ and $Q_{j}$ are the contact points of the journal and the bearing, ${\mathbf{r}}_{k}^{Q} \;(k = i,j)$ are the coordinates of the contact point on the component $k$ in the global coordinate system. The velocity of the contact point ${\dot{\mathbf{r}}}_{k}^{Q}$ is the derivative with respect to time of ${\mathbf{r}}_{k}^{Q}$.

6 $$\begin{gathered} {\mathbf{r}}_{k}^{Q} = {\mathbf{r}}_{k} + {\mathbf{A}}_{k} s{^{\prime}}_{k}^{p} + R_{k} {\mathbf{n }} \, {\kern 1pt} (k = i,j) \hfill \\ {\dot{\mathbf{r}}}_{k}^{Q} = {\dot{\mathbf{r}}}_{k} + {\dot{\mathbf{A}}}_{k} s{\prime}_{k}^{p} + R{}_{k}{\dot{\mathbf{n}}}, \hfill \\ \end{gathered}$$

7 $${\dot{\mathbf{n}}} = \dot{\alpha }{\mathbf{t}}.$$

$R{}_{i}$ and $R{}_{j}$ are the radii of the journal and bearing, ($\dot{ \bullet }$) is the derivative with respect to time of quantity ($\bullet$), the unit vector ${\mathbf{n}}$ is perpendicular to its derivative ${\dot{\mathbf{n}}}$, $\alpha$ is the angle between ${\mathbf{e}}$ and x-axis (see Fig. 2).

8 $$\alpha = \tan^{ - 1} \frac{{e_{y} }}{{e_{x} }}.$$

Derivation of the formula (8) with respect to time,

9 $$\dot{\alpha } = \frac{{\dot{e}_{y} e_{x} - e_{y} \dot{e}_{x} }}{{e^{2} }}.$$

The relative normal velocity $\dot{\delta }_{n}$ and tangential velocity $v_{t}$ at the collision point Q are expressed as,

10 $$\dot{\delta }_{n} = ({\dot{\mathbf{r}}}_{j}^{Q} - {\dot{\mathbf{r}}}_{i}^{Q} )^{{\mathbf{T}}} {\mathbf{n}},$$

11 $$v_{t} = ({\dot{\mathbf{r}}}_{j}^{Q} - {\dot{\mathbf{r}}}_{i}^{Q} )^{{\mathbf{T}}} {\mathbf{t}}.$$

$\dot{\delta }_{n}$ and $v_{t}$ are the quantity that the relative velocity is projected to the normal and tangential direction of the collision plane, as shown in Fig. 2.

**Figure 3:**
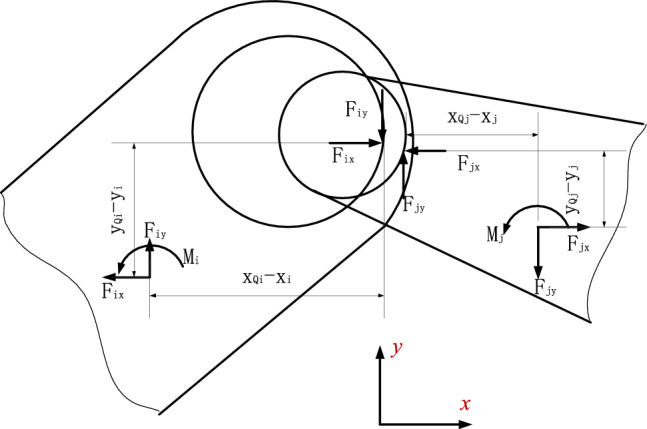
Contact force at the contact point.

At the contact point Q, the normal force ${\mathbf{F}}_{{\mathbf{N}}}$ and the tangential force ${\mathbf{F}}_{{\mathbf{T}}}$ are shown in the formula (46) and (50) respectively, transfers these forces to the center of gravity of component i and component j, as shown in Fig. 3, the forces and moments are,

12 $${\mathbf{F}}_{i} {\mathbf{ = F}}_{{\mathbf{N}}} {\mathbf{ + F}}_{{\mathbf{T}}} ,$$

13 $${\mathbf{M}}_{i} = - (x_{Qi} - x_{i} ){\mathbf{F}}_{iy} - (y_{Qi} - y_{i} ){\mathbf{F}}_{ix} ,$$

14 $${\mathbf{F}}_{j} {\mathbf{ = }} - {\mathbf{F}}_{i} ,$$

15 $${\mathbf{M}}_{j} = (x_{Qj} - x_{j} ){\mathbf{F}}_{jy} - (y_{Qj} - y_{j} ){\mathbf{F}}_{jx} .$$

The forces and moments in formula (12), (13), (14) and (15) are used as the generalized force in formula (50).

### Modeling translational joint with clearance

**Figure 4:**
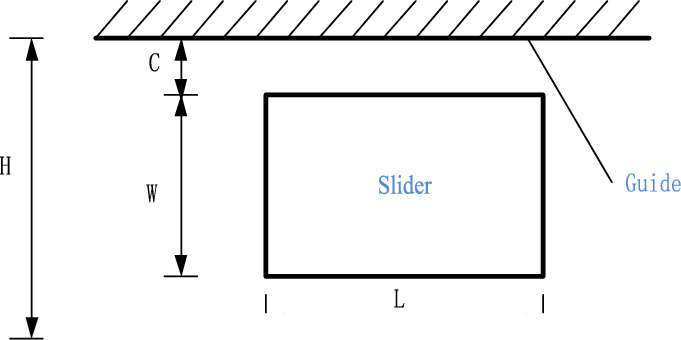
Clearance translational model.

Figure 4 is an example of translational joint with clearance, C is the distance between the surface of the rail and the slider, L is the length of the slider, W is the width of the slider, and the width of the rail is H. The clearance of the translational joint can be expressed as,

16 $$C = \frac{H - W}{2}.$$

The collision force between the slider and the guide rail of the clearance translation joint turns the constraint of the translational joint into a forced constraint, which is introduced into the dynamic equation of the mechanical system as an external force.

**Figure 5:**
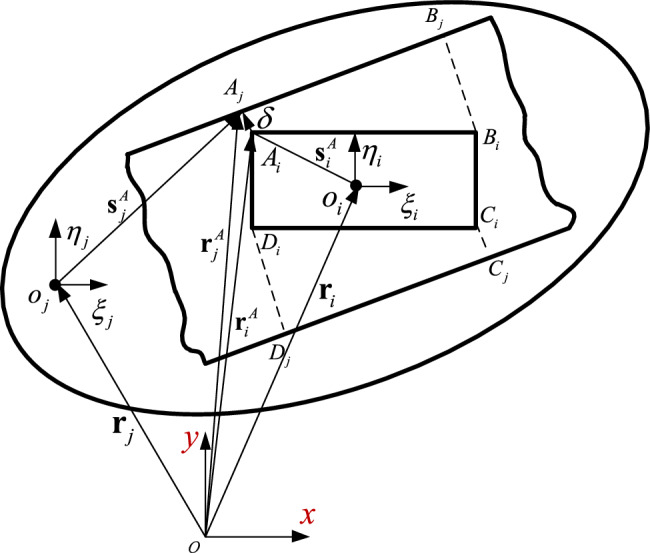
Clearance translational model in multibody system.

The model of translational clearance joint in a multibody system is shown in Fig. 5, the slider is the component i, the guide is the component j, O_i_ and O_j_ are the centroids of component i and j, respectively. Assuming ${\mathbf{t}}$ is the vector from point B_*j*_ to A_*j*_ on the guide surface, the expression is,

17 $${\mathbf{t}} = {\mathbf{s}}_{{B_{j} }} - {\mathbf{s}}_{{A_{j} }} ,$$

The vector connecting the slider point A_*i*_ to the point A_*j*_ on the rail surface is defined as,

18 $${{\varvec{\updelta}}} = {\mathbf{r}}_{j}^{A} - {\mathbf{r}}_{i}^{A} .$$

The vector δ has the same direction as the normal vector ${\mathbf{n}}$ of the guide surface, and is perpendicular to the tangential vector,

19 $${\mathbf{n}} = [t_{y} - t_{x} ]^{{\mathbf{T}}} .$$

For the contact between the slider and the guide, the vectors ${{\varvec{\updelta}}}$ and ${\mathbf{n}}$ are parallel but in opposite directions. Therefore, the penetration condition between the slider and the guide expresses as,

20 $${\mathbf{n}}^{{\mathbf{T}}} {{\varvec{\updelta}}} < 0.$$

The penetration depth of point A_*i*_ is,

21 $$\delta = \sqrt {{{\varvec{\updelta}}}^{{\mathbf{T}}} {{\varvec{\updelta}}}} .$$

${{\varvec{\updelta}}}^{{\mathbf{T}}}$ is the transpose of the vector ${{\varvec{\updelta}}}$.

The impact velocity required to calculate the contact force is obtained by differentiating Eq. (18) with respect to time,

22 $${\dot{\mathbf{\delta }}} = {\dot{\mathbf{r}}}_{j} + {\dot{\mathbf{A}}}_{j} s{^{\prime}}_{j}^{p} {\kern 1pt} {\kern 1pt} - {\dot{\mathbf{r}}}_{i} - {\dot{\mathbf{A}}}_{i} s{\prime}_{i}^{p} {\kern 1pt} {\kern 1pt} .$$

**Figure 6:**
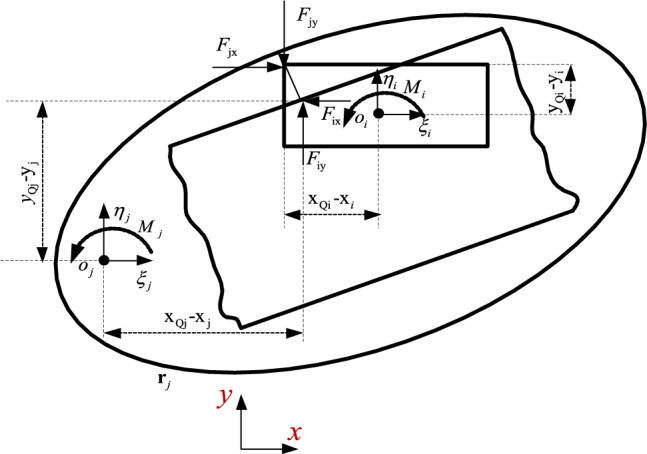
Contact force between slider and guide.

When contact occurs between the slider and the guide surface, the normal force and the tangential force act on the contact surface. The forces and moments acting on the center of mass of the components (see Fig. 6) are,

23 $${\mathbf{F}}_{i} {\mathbf{ = F}}_{{\mathbf{N}}} {\mathbf{ + F}}_{{\mathbf{T}}} ,$$

24 $${\mathbf{M}}_{i} = (x_{Qi} - x_{i} ){\mathbf{F}}_{iy} - (y_{Qi} - y_{i} ){\mathbf{F}}_{ix} ,$$

25 $${\mathbf{F}}_{j} {\mathbf{ = }} - {\mathbf{F}}_{i} ,$$

26 $${\mathbf{M}}_{j} = (x_{Qj} - x_{j} ){\mathbf{F}}_{jy} - (y_{Qj} - y_{j} ){\mathbf{F}}_{jx} ,$$

When the slider is in contact with the guide, it is assumed the contact between the spherical surface and the plane. The contact force model with hysteresis damping factor is adopted, see formula (46), the contact radius of curvature of contact point is assumed to be a small amount Rc, the equivalent stiffness is calculated by the formula $K = \frac{4}{{3(\sigma_{i} + \sigma_{j} )}}\left[ {\frac{{R_{i} R_{j} }}{{R_{i} - R_{j} }}} \right]^{{\tfrac{1}{2}}}$, where the variables $\upsilon_{k}$ and ${E}_{k}$ represent the Poisson’s ratio and Young’s modulus of the object.

## Contact force model

### Modified normal contact force model

Lankarani and Nikravesh^12^ assume that the coefficient of restitution ${C}_{e}\approx 1$, and get formula $D = \frac{{3K(1 - C_{e}^{2} )\delta^{n} }}{{4\dot{\delta }^{( - )} }}$ Since the coefficient of restitution in the simulation of actual collisions in mechanical engineering generally ranges from 0.4 to 0.8, it needs to be established a modified continuous contact force model, which is closer to the actual collision. The derivation process of the relationship between the damping coefficient and the coefficient of restitution in this modified force model is as follows.

**Figure 7:**
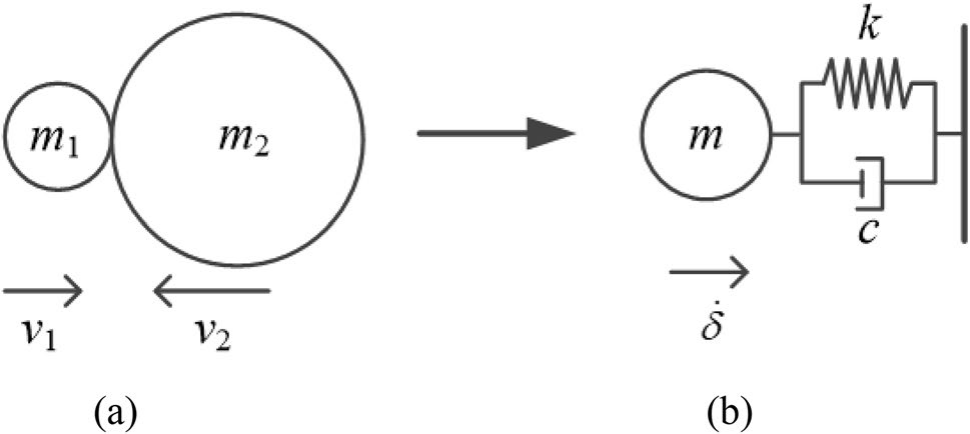
(**a**) Contact between two colliders; (**b**) Equivalent model.

The collision between two bodies (as shown in Fig. 7a) can be effectively modeled as a single degree of freedom system (as shown in Fig. 7b), the initial deformation is $\delta^{( - )} = 0$, the initial deformation velocity is $\dot{\delta }^{( - )} = v_{1}^{( - )} - v_{2}^{( - )}$, the equation of motion of the system is,

27 $$m\ddot{\delta } + c\dot{\delta } + k\delta^{n} = 0,$$

where $m$ is the quality of the equivalent system ($m = m_{1} m_{2} /(m_{1} + m_{2} )$), $c$ is the damping coefficient, and $k$ represents the equivalent stiffness.

Contact force includes spring force and damping force. According to the damping force proposed by Hunt and Crossley^11^, the expression of contact force is,

28 $$F_{N} = K\delta^{n} + \xi \delta^{n} \dot{\delta }.$$

In the process of contact collision, the simplest way to quantify the energy loss is to use the coefficient of restitution. $T^{( - )}$ and $T^{( + )}$ represent the kinetic energy value of the two collision bodies at the contact start time t^(-)^ and the contact end time t^(+)^ respectively. The energy balance expression is as follows,

29 $$\Delta E = T^{( - )} - T^{( + )} = \frac{1}{2}m(\dot{\delta }^{( - )} )^{2} - \frac{1}{2}m(\dot{\delta }^{( + )} )^{2} .$$

The coefficient of restitution represents the ratio between the velocity after the collision and the velocity before the collision of the two collision bodies, the expression is as follows,

30 $$c_{e} = - \frac{{\dot{\delta }^{( + )} }}{{\dot{\delta }^{( - )} }},$$

$\dot{\delta }^{( - )}$ and $\dot{\delta }^{( + )}$ and are the relative approach velocity at the beginning of the collision and the relative separation velocity at the end of the collision, respectively.

The coefficient of restitution expression (30) is put into formula (29),

31 $$\Delta E = \frac{1}{2}m(1 - c_{e}^{2} )(\dot{\delta }^{( - )} )^{2} ,$$

The expression of the energy balance from the beginning to the end of the compression phase is,

32 $$T^{( - )} = T^{(m)} + U^{(m)} .$$

$T^{(m)}$ is the kinetic energy at the end of the compression phase, $U^{(m)}$ is the maximum strain energy, which equals the work done by the contact force from the zero deformation state to the maximum deformation state. Assuming the contact force is the Hertz contact force $K\delta^{n}$, the formula for the energy balance at this stage is,

33 $$\frac{1}{2}m(\dot{\delta }^{( - )} )^{2} = \frac{1}{2}m(\dot{\delta }_{{^{m} }} )^{2} + \int_{0}^{{\delta_{m} }} {K\delta^{n} } d\delta .$$

$\dot{\delta }_{{^{m} }}$ represents the velocity at the end of the compression phase, which is zero at the time.

Simplify the formula (33),

34 $$(\dot{\delta }^{( - )} )^{2} = \left( {\frac{2K}{{m(n + 1)}}} \right)\delta_{m}^{n + 1} .$$

Repeating the above process, the velocity at any moment of the compression phase $\dot{\delta }$, can be obtained, $\frac{1}{2}m(\dot{\delta }^{( - )} )^{2} = \frac{1}{2}m(\dot{\delta })^{2} + \int_{0}^{\delta } {K\delta^{n} } d\delta$, that is,

35 $$\dot{\delta }^{2} = (\dot{\delta }^{( - )} )^{2} - \frac{{2K\delta^{n + 1} }}{(n + 1)m} = (\dot{\delta }^{( - )} )^{2} \left( {1 - \left( {\frac{\delta }{{\delta_{m} }}} \right)^{n + 1} } \right).$$

The energy loss can be obtained through the work done by the damping force component, as follows,

36 $$\Delta E = \oint {D\dot{\delta }d\delta = \oint {\xi \delta^{n} } } \dot{\delta }d\delta .$$

During the compression phase, the relationship between deformation velocity $\dot{\delta }$ and deformation $\delta$ is as follows,

37 $$\dot{\delta } = \dot{\delta }^{( - )} \sqrt {1 - (\frac{\delta }{{\delta_{m} }})^{n + 1} } .$$

Taking into account the recovery coefficient during the recovery phase, the relationship between the deformation speed and the deformation during the recovery period is as follows,

38 $$\dot{\delta } = \dot{\delta }^{( + )} \sqrt {1 - (\frac{\delta }{{\delta_{m} }})^{n + 1} } .$$

Substituting Eqs. (37) and (38) into (36), yields

39 $$\begin{gathered} \Delta E = \Delta E_{1} + \Delta E_{2} = \int_{0}^{{\delta_{\text{m}} }} {\xi \delta^{n} \dot{\delta }^{( - )} \sqrt {1 - (\frac{\delta }{{\delta_{\text{m}} }})^{n + 1} } } d\delta \hfill \\ + \int_{0}^{{\delta_{\text{m}} }} {\xi \delta^{n} \left| {\dot{\delta }^{( + )} } \right|} \sqrt {1 - (\frac{\delta }{{\delta_{\text{m}} }})^{n + 1} } d\delta . \hfill \\ \end{gathered}$$

The first term $\Delta E_{1}$ on the right side of formula (39) is the energy loss caused by the damping force during the compression, and the second term $\Delta E_{2}$ represents the energy dissipated during the recovery.

40 $$\Delta E = \frac{{2\xi (\dot{\delta }^{( - )} + \left| {\dot{\delta }^{( + )} } \right|)\delta_{\text{m}}^{n + 1} }}{3(n + 1)} = \frac{{2\xi (1 + c_{e} )\dot{\delta }^{( - )} \delta_{\text{m}}^{n + 1} }}{3(n + 1)}.$$

If the energy loss during the collision phase cannot be ignored, the energy balance during the beginning and the end of the compression phase can be expressed as follows,

41 $$\frac{1}{2}m(\dot{\delta }^{( - )} )^{2} = \frac{1}{2}m\dot{\delta }_{m}^{2} + K\frac{{\delta_{m}^{n + 1} }}{n + 1} + \int_{0}^{{\delta_{m} }} {\xi \delta^{n} \dot{\delta }} d\delta .$$

From formula (41), we have

42 $$\delta_{m}^{n + 1} = \frac{{m(\dot{\delta }^{( - )} )^{2} }}{{2(\frac{K}{n + 1} + \frac{{2\xi \dot{\delta }^{( - )} }}{3(n + 1)})}}.$$

Substituting formula (42) into (40), we can obtain a simplified formula,

43 $$\Delta E = \xi (1 + c_{e} )\dot{\delta }^{( - )} \frac{{m(\dot{\delta }^{( - )} )^{2} }}{{(3K + 2\xi \dot{\delta }^{( - )} )}}.$$

Combining Eqs. (43) and (31), the damping coefficient is approximately expressed by the elastic stiffness, the coefficient of restitution as,

44 $$\xi = \frac{{3K(1 - c_{e} )}}{{2c_{e} \dot{\delta }^{( - )} }}.$$

From formula (44), the following relationship between and can be obtained as,

45 $$\begin{gathered} c_{e} = 1 \to \xi = 0 \hfill \\ c_{e} = 0 \to \xi = \infty . \hfill \\ \end{gathered}$$

From the analysis of (44) and (45), it can be concluded that for a completely elastic contact, the damping coefficient is zero and the restitution coefficient is 1. When the contact is pure plastic, the damping coefficient is infinite and the restitution coefficient is 0, which is reasonable from a physical point of view.

The expression of the damping coefficient given in Eq. (44) is brought into Eq. (28), and the expression of the modified normal contact force is described as,

46 $$F_{N} = K\delta^{n} + \frac{{3K\delta^{n} (1 - c_{e} )}}{{2c_{e} }}\frac{{\dot{\delta }}}{{\dot{\delta }^{( - )} }}.$$

The first term on the right side of the equation is the elastic deformation force, and the second term is the damping force. *K* is the contact stiffness coefficient $K = \frac{4}{{3(\sigma_{i} + \sigma_{j} )}}\left[ {\frac{{R_{i} R_{j} }}{{R_{i} - R_{j} }}} \right]^{{\tfrac{1}{2}}}$, $\sigma_{k} = \frac{{1 - \upsilon_{k}^{2} }}{{E_{k} }}{\kern 1pt} {\kern 1pt} {\kern 1pt} {\kern 1pt} {\kern 1pt} {\kern 1pt} {\kern 1pt} {\kern 1pt} (k = i,j)$ is determined by the elastic modulus *E*, Poisson’s ratio $\upsilon_{k}$, and contact radius *R* of the two contact bodies,

Equation (46) is consistent with the contact force model mentioned in reference^47,48^, which was obtained by fitting the deformation velocity and deformation relationship curve, when $\beta$ = 5/2^47^, the contact force model was derived, and the value of index $n$ = 3/2. However, the value $n$ in $\sigma_{k} = \frac{{1 - \upsilon_{k}^{2} }}{{E_{k} }}\,\left( {k = i,j} \right)$ the contact force model deduced of this paper is unlimited, which has more general significance. Literature^47^ also just verified the correctness of our formula.

**Figure 8:**
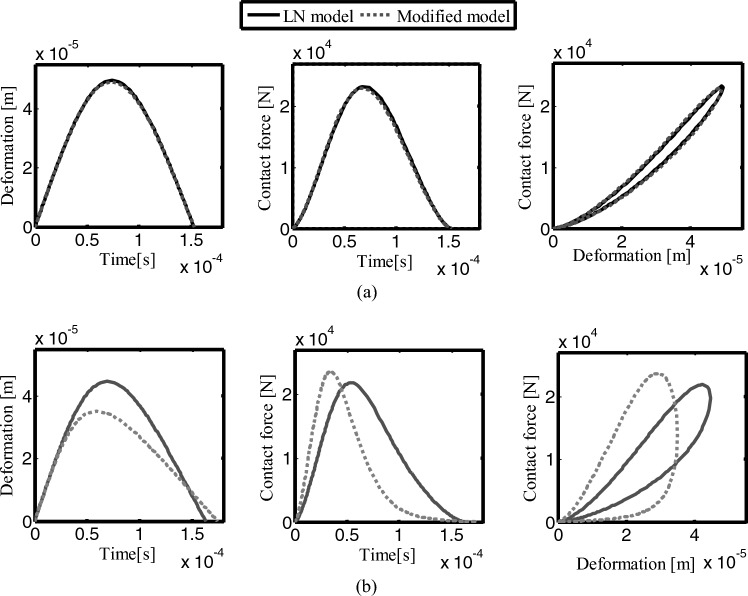
Comparison of the contact process between the modified contact model and the LN model.

Figure 8 shows the collision process compared between the modified contact force model and the LN model. In Fig. 8a, the coefficient of restitution is 0.9, and the deformation and the contact force calculated by the two models are relatively close. In Fig. 8b, the coefficient of restitution is 0.4. It can be clearly seen that compared to the LN model, the modified contact force model consumes more energy and has a larger force–deformation hysteresis loop. Therefore, the modified contact force model can better describe the energy dissipation with different restitution coefficients, and the contact force model has better application prospects.

**Figure 9:**
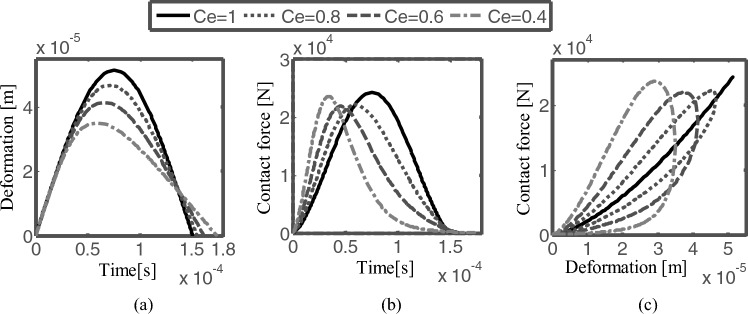
The contact process of the modified contact model with different restitution coefficients (v_0_ = 1 m/s).

Figure 9 shows the collision process of the contact force model with different restitution coefficients at the initial collision velocity ${v}_{0}=1m/s$, and the clearance between the journal and the bearing is 0.5 mm. Figure 9a is the relationship between deformation and time, Fig. 9b is contact force and time, and Fig. 9c is contact force and deformation. When the coefficient of restitution is larger, the time of the compression phase is longer, and the contact deformation is larger, the duration of the recovery phase and the total contact are shorter. The relationship between force and deformation shows hysteresis damping characteristics, reflecting the energy dissipation in the contact process. When the coefficient of restitution is small, the energy loss is greater. The modified contact model can accurately describe the energy loss with different restitution coefficients.

When the coefficient of restitution is equal to 1, it is the Hertz contact force, there is no energy loss during the contact. When the coefficient of restitution is relatively high (i.e. close to 1), both models can reflect the energy dissipation of the contact process between the journal and bearing. When the coefficient of restitution is low, the contact process is different, the modified contact force model can better reflect the energy dissipation of the contact process, which reflects the greater energy dissipation and is more in line with the actual situation.

It is worth mentioning that only the modified contact force model and LN model are compared in this paper. Other models can be referred to some literatures^48–50^.

### Tangential-friction force models

The LuGre model^51,52^ was proposed by Canudas de Wit et al. This model is a model that can accurately predict the friction characteristics and has a better dynamic compensation effect for the friction, it can capture the Stribeck and static friction effects. Although the implementation of LuGre friction in revolute joints with clearance cannot accurately simulate the physical friction phenomenon, it is closer to the friction phenomenon than the classical and modified Coulomb’s law. For more details about the calculation implementation of LuGre friction law in revolute joints can be found in^27^.

**Figure 10:**
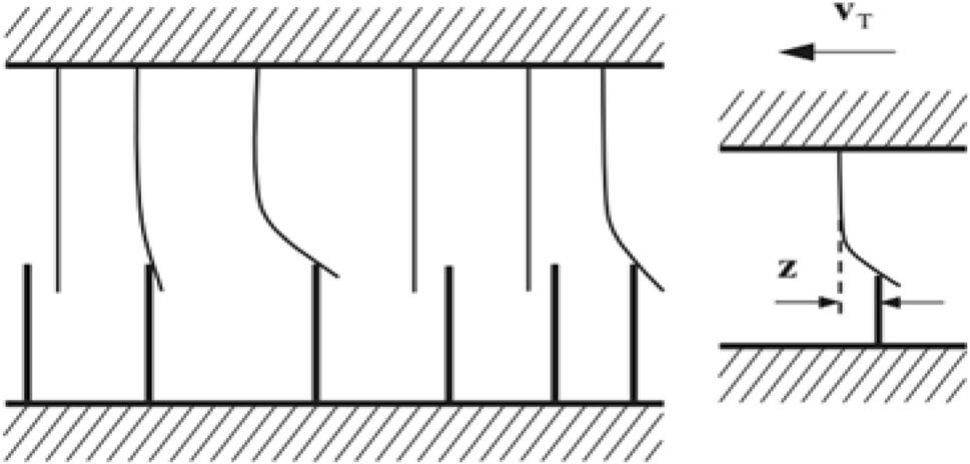
LuGre physical model.

To quantify the average bristle deflection, an internal state variable z needs to be introduced, as shown in Fig. 10. The LuGre friction is as follows,

47 $${\mathbf{F}}_{{\mathbf{T}}} = \mu {\mathbf{F}}_{{\mathbf{N}}} .$$

The instantaneous coefficient of friction $\mu$ is given as,

48 $$\mu = \sigma_{0} {\mathbf{z}} + \sigma_{1} {\kern 1pt} {\kern 1pt} ({\mathbf{v}}_{{\mathbf{T}}} )\frac{{d{\mathbf{z}}}}{dt}{\kern 1pt} + \sigma_{2} {\mathbf{v}}_{{\mathbf{T}}} ,$$

49 $$\begin{gathered} {\mathbf{z}} = \frac{{v_{T} }}{{\left| {v_{T} } \right|}} \times \frac{{\mu_{k} + (\mu_{s} - \mu_{k} )e^{{ - \left| {\frac{{v_{T} }}{{v_{S} }}} \right|^{2} }} }}{{\sigma_{0} }} \hfill \\ \frac{{d{\mathbf{z}}}}{dt}{\kern 1pt} = \left( {1 - \frac{{\sigma_{0} }}{{\mu_{k} + (\mu_{s} - \mu_{k} )e^{{ - \left| {\frac{{v_{T} }}{{v_{S} }}} \right|^{2} }} }}{\mathbf{z}} \cdot {\text{sgn}} ({\mathbf{v}}_{{\mathbf{T}}} )} \right){\mathbf{v}}_{{\mathbf{T}}} , \hfill \\ \end{gathered}$$

where $\sigma_{0}$ represents the stiffness of the bristles, $\sigma_{1} {\kern 1pt} {\kern 1pt}$ is the microscopic damping coefficient, μ_*k*_ is dynamic friction coefficient, μ_*s*_ is static friction coefficient. This friction coefficient $\mu$ combines three parts, pre-sliding friction $\sigma_{0} {\mathbf{z}}$, Stribeck friction $\sigma_{1} {\kern 1pt} {\kern 1pt} \frac{{d{\mathbf{z}}}}{dt}{\kern 1pt}$ and viscous friction $\sigma_{2} {\mathbf{v}}_{{\mathbf{T}}}$. The above parameters are listed in Table 2.

## Dynamic modeling of mechanism with clearance joint

In this study, the modified normal contact force model is used to calculate the normal contact force, and the LuGre friction force model is used to calculate the tangential contact force. The force generated during the contact of the clearance joint is introduced as an external force into the motion equation^53^. Since the motion equations are strongly nonlinear, they can only be solved by numerical methods, Since Baumgarte Stability Method (BSM) is easy to implement on a computer, this paper uses this method to control the position and velocity constraint violations caused by the direct integration method.

50 $$\left[ {\begin{array}{*{20}c} {\mathbf{M}} & {{{\varvec{\Phi}}}_{{\mathbf{q}}}^{{\mathbf{T}}} } \\ {{{\varvec{\Phi}}}_{{\mathbf{q}}} } & {\mathbf{0}} \\ \end{array} } \right]\left\{ {\begin{array}{*{20}c} {\ddot{\mathbf{q}}} \\ {{\varvec{\uplambda}}} \\ \end{array} } \right\} = \left\{ {\begin{array}{*{20}c} {\mathbf{g}} \\ {{{\varvec{\upgamma}}} - 2\alpha {\dot{\mathbf{\Phi }}} - \beta^{2} {{\varvec{\Phi}}}} \\ \end{array} } \right\}$$

where ${\mathbf{M}}$ is the mass matrix including the mass and moment of inertia of system components. ${\ddot{\mathbf{q}}}$ and ${{\varvec{\Phi}}}_{{\mathbf{q}}}^{{\mathbf{T}}}$ are acceleration vector and Jacobian matrix respectively. ${{\varvec{\uplambda}}}$ is the Lagrangian multiplier vector. ${\mathbf{g}}$ is the applied external load vector. ${\mathbf{\gamma = }} - {\mathbf{(\Phi }}_{{\mathbf{q}}} {\dot{\mathbf{q)}}}_{{\mathbf{q}}} {\dot{\mathbf{{q} - 2\Phi }}}_{{{\mathbf{q}}t}} {\dot{\mathbf{{q} - \Phi }}}_{tt}$.

The Lagrangian multiplier vector ${{\varvec{\uplambda}}}$ and the generalized acceleration vector ${\ddot{\mathbf{q}}}$ can be gotten from Eq. (50),

51 $$\left[ {\begin{array}{*{20}c} {\ddot{\mathbf{q}}} \\ {{\varvec{\uplambda}}} \\ \end{array} } \right] = \left[ {\begin{array}{*{20}l} \begin{gathered} [{\mathbf{M}}^{ - 1} - {\mathbf{M}}^{ - 1} {{\varvec{\Phi}}}_{{\mathbf{q}}}^{{\mathbf{T}}} ({{\varvec{\Phi}}}_{{\mathbf{q}}} {\mathbf{M}}^{ - 1} {{\varvec{\Phi}}}_{{\mathbf{q}}}^{{\mathbf{T}}} )^{ - 1} {{\varvec{\Phi}}}_{{\mathbf{q}}} {\mathbf{M}}^{ - 1} ]{\mathbf{g}} \hfill \\ + {\mathbf{M}}^{ - 1} {{\varvec{\Phi}}}_{{\mathbf{q}}}^{{\mathbf{T}}} ({{\varvec{\Phi}}}_{{\mathbf{q}}} {\mathbf{M}}^{ - 1} {{\varvec{\Phi}}}_{{\mathbf{q}}}^{{\mathbf{T}}} )^{ - 1} [{{\varvec{\upgamma}}} - 2\alpha {\dot{\mathbf{\Phi }}} - \beta^{2} {{\varvec{\Phi}}}] \hfill \\ \end{gathered} \\ \begin{gathered} ({{\varvec{\Phi}}}_{{\mathbf{q}}} {\mathbf{M}}^{ - 1} {{\varvec{\Phi}}}_{{\mathbf{q}}}^{{\mathbf{T}}} )^{ - 1} {{\varvec{\Phi}}}_{{\mathbf{q}}} {\mathbf{M}}^{ - 1} {\mathbf{g}} \hfill \\ - ({{\varvec{\Phi}}}_{{\mathbf{q}}} {\mathbf{M}}^{ - 1} {{\varvec{\Phi}}}_{{\mathbf{q}}}^{{\mathbf{T}}} )^{ - 1} [{{\varvec{\upgamma}}} - 2\alpha {\dot{\mathbf{\Phi }}} - \beta^{2} {{\varvec{\Phi}}}] \hfill \\ \end{gathered} \\ \end{array} } \right].$$

Baumgarte proposed a criterion to select the coefficient $\alpha$, $\beta$. Kim et al.^54^ think that when using a constant step size, the feedback parameter $\alpha = 1/\Delta t$ and $\beta = \sqrt 2 /\Delta t$, where $\Delta t$ is the integration step, this method is simple and very effective for computer implementation.

The internal collision between journal and bearing will generally occur during the operation of the mechanism with clearance joint. One of the most critical points in the dynamic simulation of collision multibody system is the detection of precise collision instant, so it is necessary to monitor the movement state of the mechanism with clearance joint through the relative position of the corresponding contact point between the journal and the bearing. There is a switching point of motion state in the time interval [t, t + △t], so that δ(*q*(*t*))^*T*^ δ(q(*t* + △t)) < 0, Usually, such a precise moment can be found by using Newton–Raphson method.

**Figure 11:**
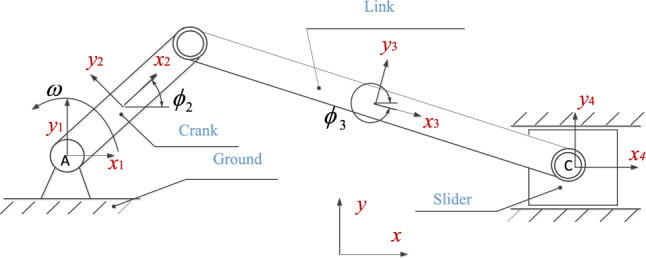
Schematic graph of slider-crank mechanism with clearance joints.

**Figure 12:**
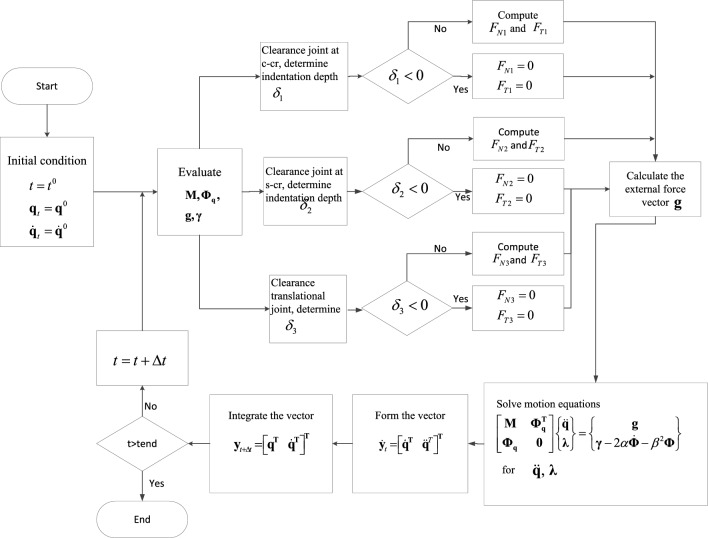
Dynamic calculation scheme of multibody mechanical system with clearance joints.

In this study, the typical crank-slider mechanism shown in Fig. 11 is taken as an example to study the dynamic response of a multi-body mechanical system with clearance. The calculation scheme for the dynamic of a mechanical system with multiple clearance joints is shown in Fig. 12. During the simulation, the initial state of the mechanism is that the crank and connecting rod are collinear, and the shaft and sleeve are concentric. The crank rotates at a constant angular velocity, the initial position and velocity of the ideal structure motion simulation are selected as the initial values. In this calculation scheme, a new vector $y$ and $\dot{y}$ is introduced, and the numerical integration algorithm becomes a first-order differential equation. After integrating the velocity and acceleration at time t, the position and velocity at the next time $t + \Delta t$ are obtained, and the time step $\Delta t$ is a known quantity. The dynamic steps for solving the crank-slider mechanism with clearance joints are as follows:

1. Starting from the initial time $t_{0}$, position ${\mathbf{q}}_{0}$ and velocity ${\dot{\mathbf{q}}}_{0}$ are given as the initial conditions.
2. Construct the constraint equation **Φ**, calculate the mass matrix ${\mathbf{M}}$, the Jacobian matrix **Φq**, the right term γ of the acceleration constraint equation, the external force vector ${\mathbf{g}}$.
3. According to the position and configuration of the system, the dynamic analysis of the system with three clearance joints is carried out at the same time, and the deformation depth δ is calculated by formulas (1), (2) and (3) and (5);
4. If the deformation depth of the clearance joint is greater than or equal to zero, use formula (46) to calculate the contact force and formula (47) to calculate the LuGre friction force. Otherwise, the normal contact force and tangential friction force are zero.
5. Transfer the contact force Eqs. (12) and (14) as external forces to the contact point of the clearance joint, the corresponding torque Eqs. (13) and (15) produced by the external force act on the connecting rod, and the external force and moment are used in the system motion equations.
6. Solve the multi-body system motion Eq. (51), get the acceleration at time t and the Lagrangian multiplier λ.
7. Assemble the generalized velocity ${\dot{\mathbf{q}}}$ and acceleration ${\ddot{\mathbf{q}}}$ at time t, expressed as a vector ${\dot{\mathbf{y}}}_{t}$, namely $${\dot{\mathbf{y}}}_{t} = \left[ {\begin{array}{*{20}c} {{\dot{\mathbf{q}}}^{{\mathbf{T}}} } & {\ddot{\mathbf{q}}^{{\mathbf{T}}} } \\ \end{array} } \right]^{{\mathbf{T}}}$$.
8. Calculate the position ${\mathbf{q}}$ and velocity ${\dot{\mathbf{q}}}$ at time t + Δt by numerical integration ${\dot{\mathbf{y}}}_{t}$.
9. Update the time variable and go to step (2), and continue to execute the program until the last moment of analysis tend.

## Experimental test rig

In this section, experimental research on the dynamic response of the slider-crank mechanism with a clearance joint was conducted, the clearance joint connects the slider and link. The purpose of the experiment is to verify the dynamic prediction model in the previous section. The slider acceleration is tested by changing the crank speed and the size of the clearance, the numerical simulation and experimental results are compared.

### Experimental test rig of the mechanism with clearance

**Figure 13:**
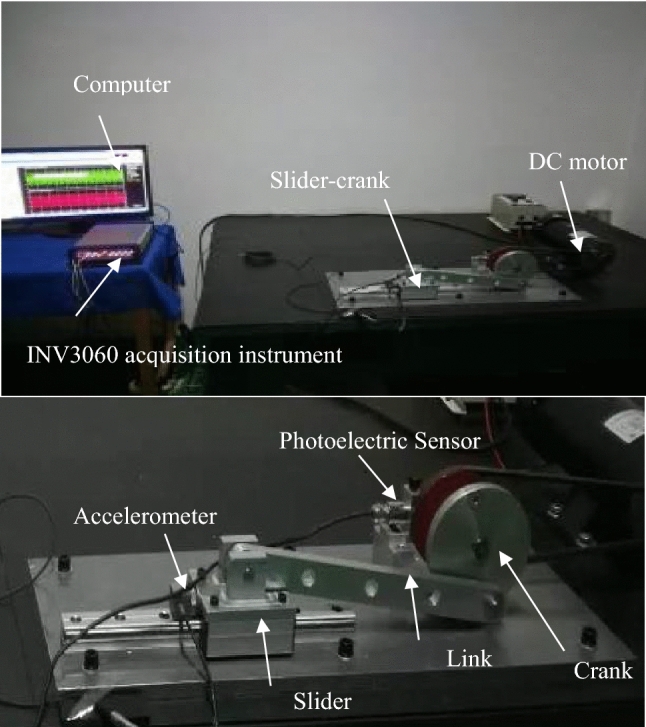
Experimental test rig for slider-crank mechanism.

**Figure 14:**
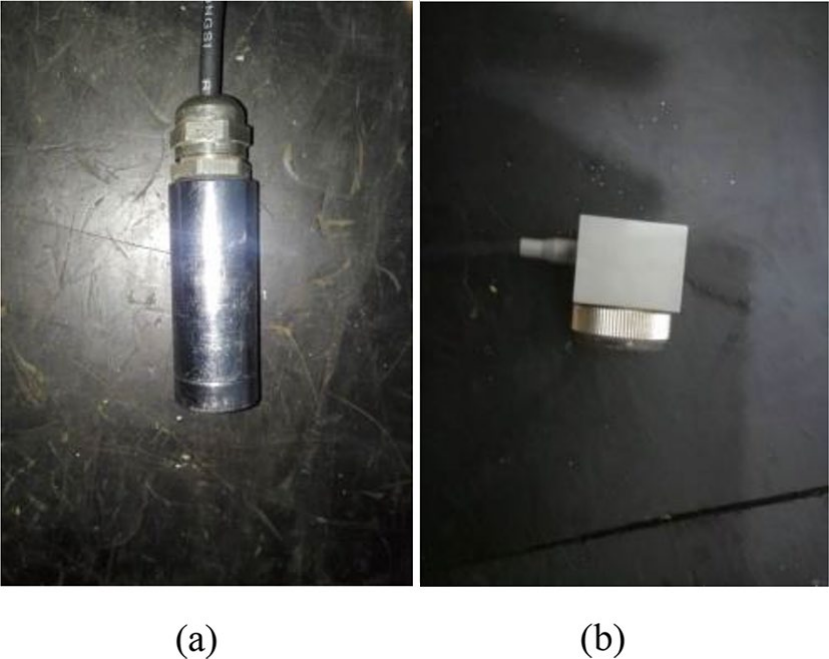
(**a**) Photoelectric sensor; (**b**) three-way acceleration sensor.

**Figure 15:**
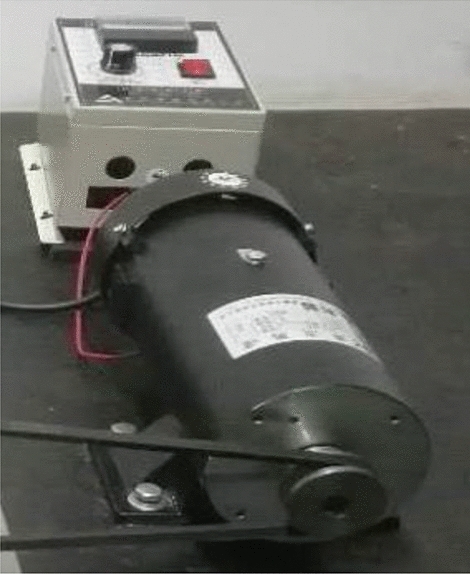
Drive motor and transmission.

To study the dynamic response of mechanical systems with clearance joints, an experimental test rig was constructed, Fig. 13 shows the experimental device, where the joint between the link and the slider has clearance, the mechanism works on a vertical plane, and the mechanical components are made of aluminum alloy. The journal is rigidly connected to the slider, and the clearance between the journal and the bearing can be changed by changing the journal, the other revolute joints use needle bearings with minimal radial clearance, and oil these joints to reduce their friction as close as possible to the ideal joint. The slider is a linear translation bearing with precision preload system with zero clearance. The motion characteristic of the slider of the test platform are measured by acceleration sensor, the three-way acceleration sensor is shown in Fig. 14. A 550W DC variable speed motor (shown in Fig. 15) is selected as the power source, the rotation speed is 1800 rpm. The photoelectric sensor (shown in Fig. 14) is used to detect the rotation speed and angle of the crank, and the three-way acceleration sensor is used to detect the slider acceleration. The data acquisition equipment is INV3060 24-bit network intelligent acquisition instrument (shown in Fig. 13), which plays a role of connecting sensors and computer, and it is the key component to ensure the working performance of the whole system.

### Comparison of experimental and numerical simulation results of the mechanism with clearance

This section mainly conducts numerical and experimental studies on the dynamic performance of the slider-crank mechanism with a clearance joint. The experimental test rig allows two adjustable parameters: radial clearance and crank speed. Table 1 shows the physical properties of the experimental mechanism. Table 2 lists the parameters used in the dynamic simulation.

**Table 1:** Physical quantities of the slider-crank mechanism.

Body	Length (m)	Mass (kg)	Moment of inertia (kg × m^2^)
Crank	0.05	1.5261	3.596e-003
Link	0.3	0.6115	0.006100
Slider	–	1.0205	–

**Table 2:** Dynamics simulation parameters.

Parameter	Value	Parameter	Value
Crank speed	100/150/200/250 rpm	Baumgarte-α	1,000,000
Young’s modulus	70 GPa	Baumgarte-β	1,410,000
Poisson’s ratio	0.33	Dynamic friction coefficient,$\mu_{k}$	0.01
Bearing radius	12 mm	Static friction coefficient,$\mu_{s}$	0.01
journal radius	11.5 mm	Bristle stiffness coefficient,$\sigma_{0}$	1e5N/m
CoT	0.5 mm	Bristle damping coefficient,$\sigma_{1}$	400Ns/m
Coefficient of restitution	0.55	Viscous friction coefficient $\sigma_{2}$	0
Integration step	0.000001 s	Stribeck velocity, *v*_s_	0.001 m/s

In the modeling of the collision phenomenon of the multi-body system, the selection of the friction coefficient and the restitution coefficient is very important and affects the calculation results. The value of the restitution coefficient depends on the impact velocity, for relatively low or moderate impact velocities, the value does not change significantly and can be assumed to be a constant value^55^. Some researchers^26,56–58^ adopted friction coefficient values in the range of 0.007 ~ 0.01 and the restitution coefficient is with clearance. Haroun^59^ compared the simulation and experimental results and found 0.4 ~ 0.6 to study the dynamics of mechanism that the simulation results are very close to the experimental results when the coefficient of restitution is 0.55. Therefore, in this paper, we choose the dynamic friction, static friction and restitution coefficient of 0.01, 0.01 and 0.55, respectively, to simulate the mechanism.

**Figure 16:**
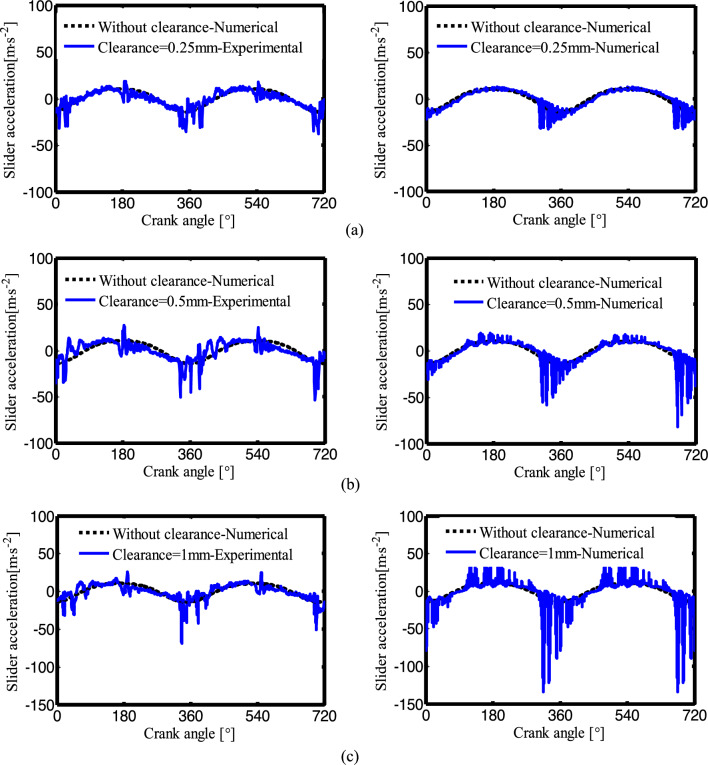
Slider acceleration of experiment and simulation when the crank speed is 150 rpm: (**a**) The radial clearance c = 0.25 mm; (**b**) c = 0.5 mm; (**c**) c = 1 mm.

Figure 16 shows slider acceleration of experiment and simulation when the crank speed is 150rpm and the radial clearance is 0.25mm, 0.5mm and 1mm, the left is experimental results, and the right is the simulation results. It can be seen from the figure, when the crank rotates to around 180° or 360°, the acceleration amplitude of the slider has obvious oscillations, the larger the clearance, the more obvious the oscillation. The maximum slider acceleration increases with the increasing of radial clearance.

**Figure 17:**
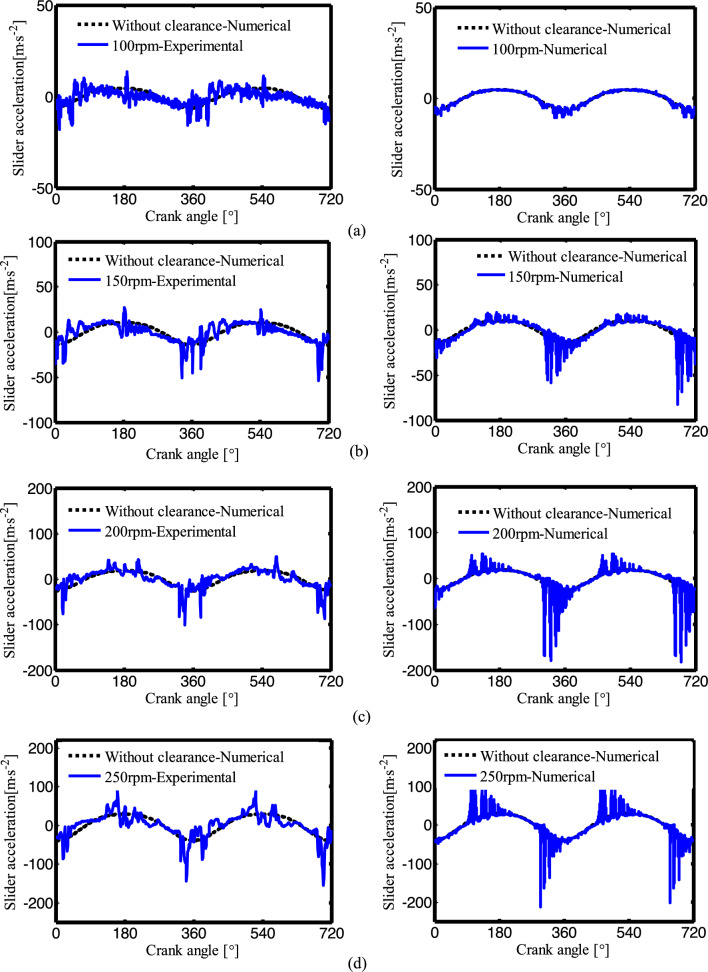
Slider acceleration of experiment and simulation when the radial clearance is 0.5 mm: (**a**) The crank speed n = 100 rpm; (**b**) n = 150 rpm; (**c**) n = 200 rpm; (**d**) n = 250 rpm.

Figure 17 shows the effect of different crank speeds on the slider acceleration response.The experimental results are shown on the left, and the simulation results are shown on the right. The crank speeds are 100 rpm, 150 rpm, 200 rpm and 250 rpm, and the radial clearance is 0.5 mm. For lower crank speeds (not more than 150 rpm), the overall motion of the crank-slider mechanism changes less, and the slider acceleration is similar to the experimental results. For higher crank speeds (more than 150 rpm), the peak value of slider acceleration increases.

In general, for lower crank speeds and smaller clearance mechanisms, the kinematic characteristics of the slider-crank mechanism are similar to those of the experimental results, for higher frequencies and larger clearances, the dynamic response changes significantly. The peak value of the experiment is smaller than the peak value of the simulation, which is because the flexibility of the joints and links are ignored in the mathematical model of the clearance joint. Comparing to the peak value of the simulation, the experimental values lag slightly, this is due to the use of preloaded linear guide slider. The agreement between the experiment and numerical simulation is verified and directions are set for subsequent research.

## Simulation and results

The simulation model of the slider-crank mechanism is shown in Fig. 11, and the dynamic analysis of the mechanism with clearance joints is carried out. The geometric parameters are shown in Table 1, and the dynamic simulation parameters are shown in Table 2.

Schemes for nonlinear dynamic analysis of mechanism with different types of clearance joints and different numbers of clearance joints:

1. Ideal mechanism;
2. Only considering the clearance at the prismatic joint D, the clearance is 0.5 mm, and the rest are ideal moving pairs;
3. Only considering the clearance at the revolute joints B and C, the clearance at the revolute joint B and C are both 0.5 mm and the other motion pairs are ideal;
4. Considering the clearance at joints B, C and D, the clearance is the same as the previous one, and the rest motion pairs are ideal.

In this section, the dynamic characteristics of the mechanism under the action of mixed clearance joints are studied. That is, the dynamic analysis of the slider-crank mechanism with two revolute joints B and C, and one prismatic joint D is carried out, as shown in Fig. 11. The driving speed is 200 rpm, and other conditions are shown in Tables 1, 2. Two complete cycles during the steady-state operation of the mechanism are selected for analysis.

**Figure 18:**
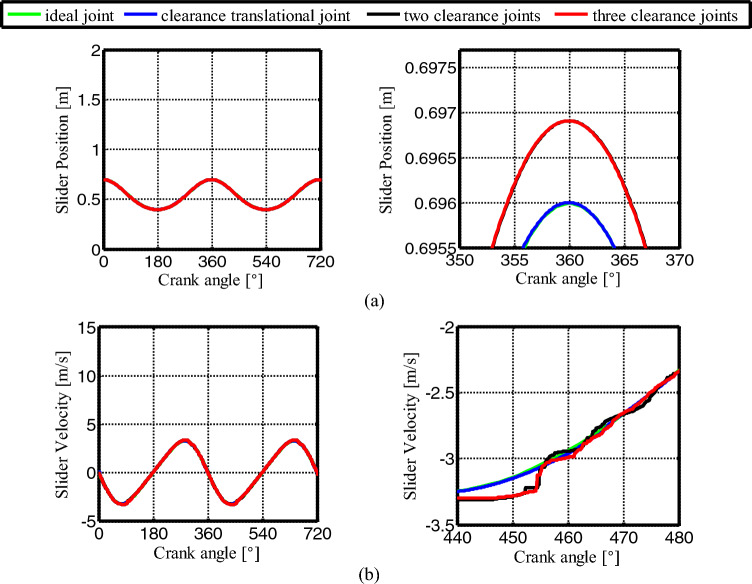
(**a**) Slider displacement for different number of clearance joint: (**b**) Slider velocity.

Figure 18 is the figure of slider displacement and velocity under four motion conditions. It can be seen from Fig. 18 that the displacement and velocity obtained under the conditions of only considering the prismatic joint, considering the revolute joint and considering the mixed the clearance joint are consistent with those of the ideal mechanism. The clearance in the joint has little effect on the displacement and velocity of the slider. However, from their enlarged figures (the figures on the right), the clearance in the revolute joint makes the displacement deviate from the ideal mechanism, and the velocity curve shows a step-like, when only considering the clearance in prismatic joint, the displacement and velocity curves are almost consistent with the ideal mechanism. Compare to the clearance in revolute joint and the one in prismatic joint, the former has much less influence on the displacement and velocity of the slider.

**Figure 19:**
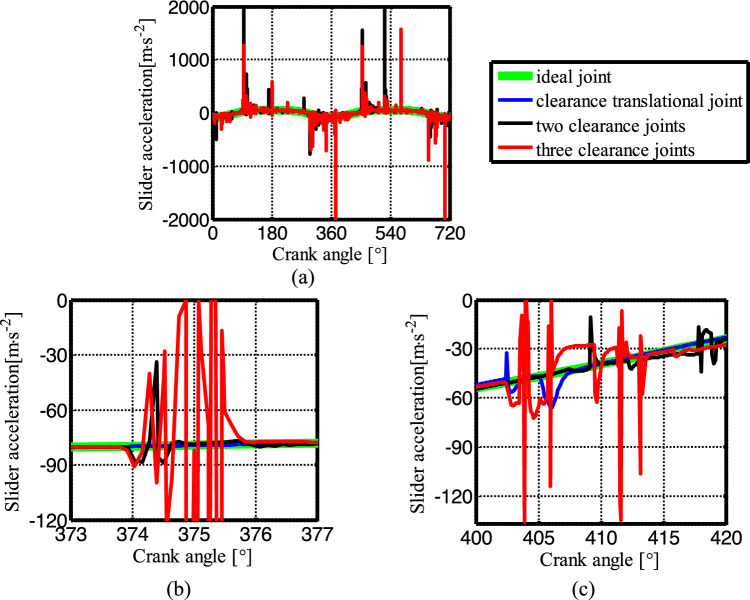
(**a**) Slider acceleration for different number of clearance joint; (**b**) detailed view between 373$^\circ$  and  377$^\circ$; (**c**) detailed view between 400$^\circ$ and 420°.

**Figure 20:**
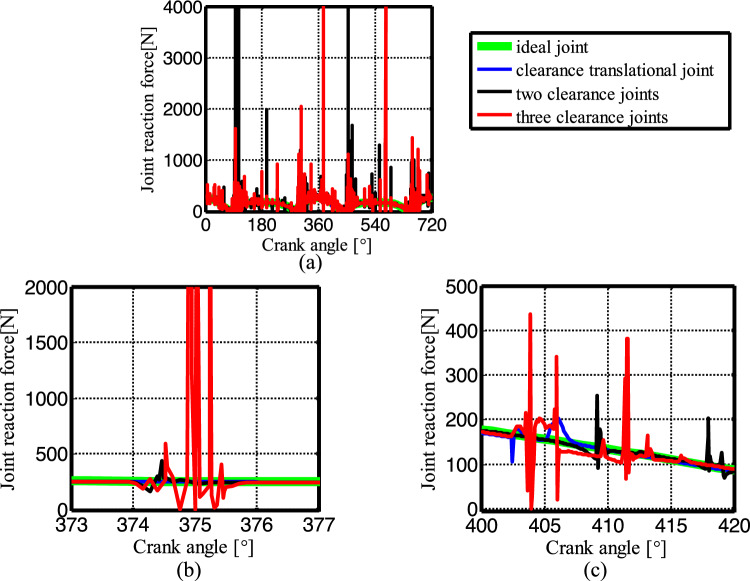
(**a**) Contact force at joint B for different number of clearance joint; (**b**) detailed view between 373$^\circ$ and 377$^\circ$; (**c**) detailed view between 400$^\circ$ and 420$^\circ$.

**Figure 21:**
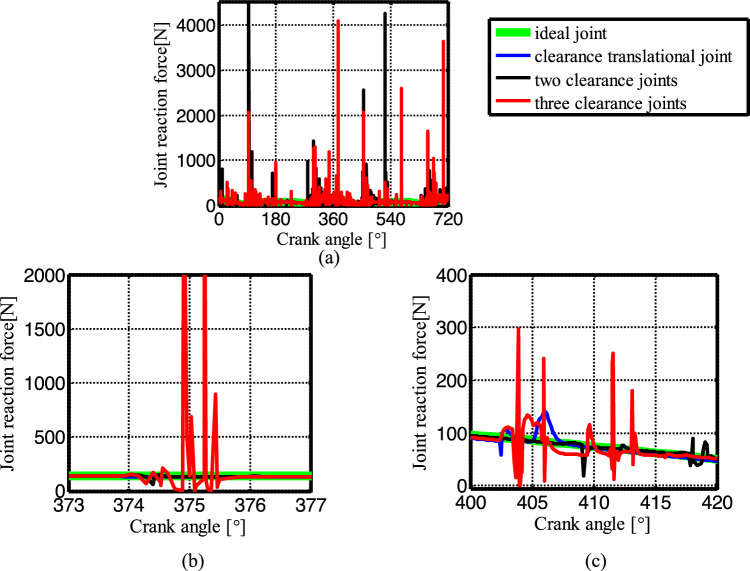
(**a**) Contact force at joint C for different number of clearance joint; (**b**) detailed view between 373$^\circ$ and 377$^\circ$; (**c**) detailed view between 400$^\circ$ and 420°.

**Figure 22:**
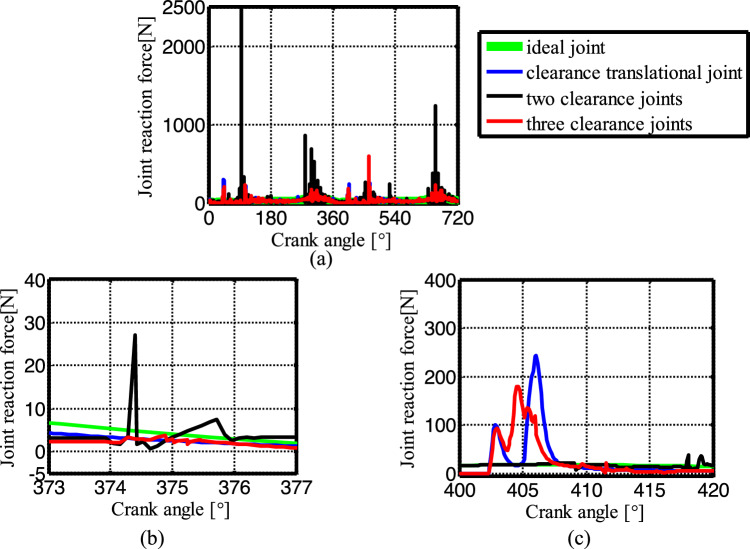
(**a**) Contact force at joint D for different numbers of clearance joint; (**b**) detailed view between 373$^\circ$ and 377$^\circ$; (**c**) detailed view between 400$^\circ$ and 420$^\circ$.

**Figure 23:**
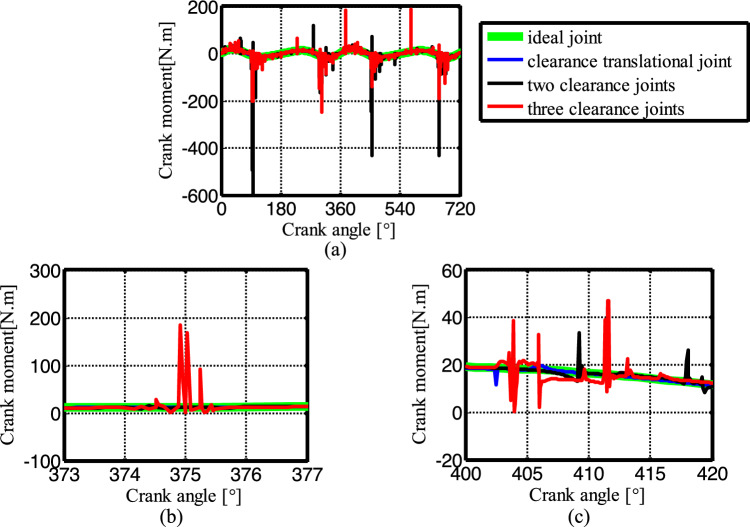
(**a**) Crank balance moment for different numbers of clearance joint; (**b**) detailed view between 373° and 77$^\circ$; (**c**) detailed view between 400$^\circ$ and 420$^\circ$.

Figure 19 shows the acceleration of the slider. The contact force at the joints B, C, and D are shown in Figs. 20, 21 and 22, respectively. As shown in Fig. 23, the crank balance moment under four different conditions is shown in Fig. 23. (b) and (c) of Figs. 19, 20, 21, 22 and 23 are partial enlargement. In general, when the mechanism considers only two clearance joints, the position of the dynamic peak values is consistent with the mechanism considering the three hybrid clearance joints, while the mechanism considering only the translational joint is similar to the ideal mechanism, and peaks appear locally.

From the enlarged figure (b) of Figs. 19, 20, 21, 22 and 23, we can see that the dynamic value of the mechanism with two revolute joints has a peak area around 374.3°, while the dynamic value of the mechanism with clearance translational joint doesn’t have one. However, the dynamic value of the mechanism with three joints has a large peak around 375°. Clearance translational joint exacerbates or slows down this vibration peak, for example, the existence of clearance joint D aggravates the contact force peaks at joints B and C, as shown by the red line in Fig. 20b, 21b, while at joint D, contact force peak reduces, see Fig. 22b. When the clearance translational joint D collides, it has a certain influence on the clearance revolute joint. From the blue line in Fig. 22c, it can be seen that the clearance joint D collides near the crank angle of 405°, the clearance joint C collides immediately, as shown in Fig. 21c, the joint C collides, it causes the joint B to collide. The dynamic peak frequency of the three clearance joints mechanism is increased relative to the one only considering the revolute joint mechanism. The joint forces at joints B, C and D generally show a downward trend. Figure 23 is the crank balance moment, which is similar to the acceleration and the contact force. The peaks of the clearance joints B, C and D also reflect in the crank balance moment.

**Figure 24:**
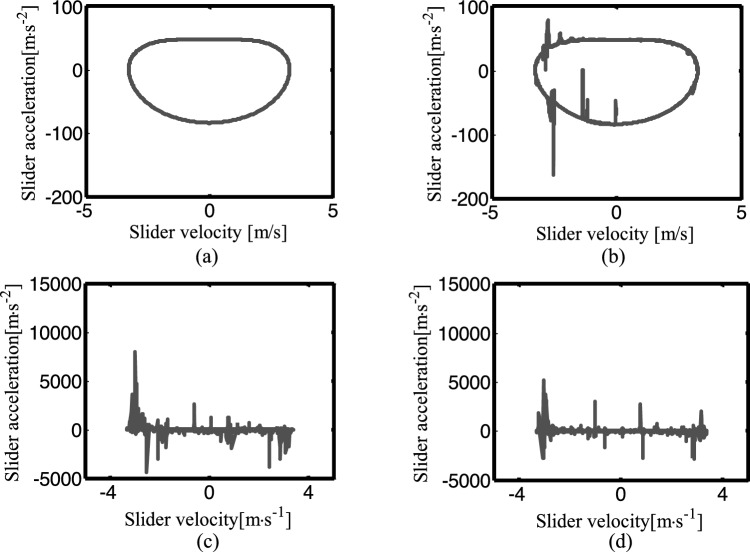
Phase diagram for different numbers of clearance joint: (**a**) an ideal mechanism; (**b**) the mechanism considering a clearance translational joint; (**c**) two revolute joints mechanism; (**d**) three clearance joints mechanism.

Figure 24 shows the phase diagram of the slider velocity and acceleration under four different conditions. Taking the data of the slider velocity and acceleration for 5 cycles after 50 crank rotation cycles. Figure 24a is an ideal mechanism diagram, Fig. 24b is the diagram of the mechanism considering a clearance translational joint, Fig. 24c is a diagram of two revolute joints mechanism, and Fig. 24d is the diagram of three clearance joints mechanism. From the figures, we can see the clearance has little effect on the velocity of the slider, but it has a great effect on the acceleration, and the acceleration has peak values. When the mechanism with only one clearance translational joint, its phase diagram shows a small number of peaks. Compared with the corresponding phase diagram of the mechanism with clearance revolute joints, it is closer to the phase diagram of the ideal mechanism. With the increase of the number of clearance joints, the acceleration amplitude and peak frequency in the phase diagram show an increasing trend.

**Figure 25:**
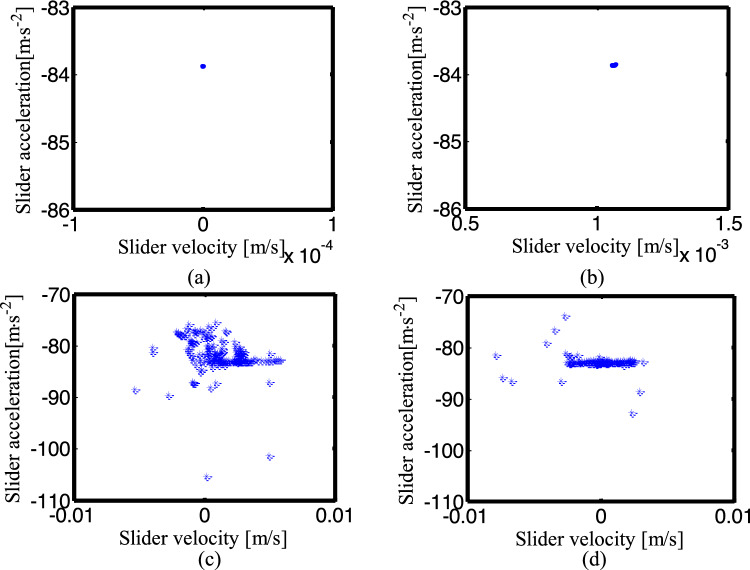
Poincaré maps for different numbers of clearance joint. (**a**) an ideal mechanism; (**b**) the mechanism considering a clearance translational joint; (**c**) two revolute joints mechanism; (**d**) three clearance joints mechanism.

Figure 25 depicts the Poincaré map for the mechanisms with different numbers of clearance joint. When the crank angle is 0°, select the data of the slider velocity and acceleration for + cycles after the crank rotates 50 cycles. Figure 25a–d is the phase diagram of the ideal mechanism, the mechanism with one clearance translational joint, the mechanism with two clearance revolute joints and the one with three clearance joints, respectively. The ideal mechanism Poincaré map is a point, which means a periodic motion. The Poincaré mapping translational joint are several concentrated points, so the motion is periodic. The Poincaré mapping points of the mechanism with two clearance revolute joints are mainly distributed from − 0.005 to 0.005 m/s in velocity, − 90 ~ 75 m/s^2^ in acceleration, it is a chaotic motion. The Poincaré map with three clearance joints illustrated in Fig. 25d, the distribution range of the points is reduced, indicating that the third clearance joint has the effect of alleviating the impact, so the range of the mapping points is reduced, the motion is chaotic.

**Figure 26:**
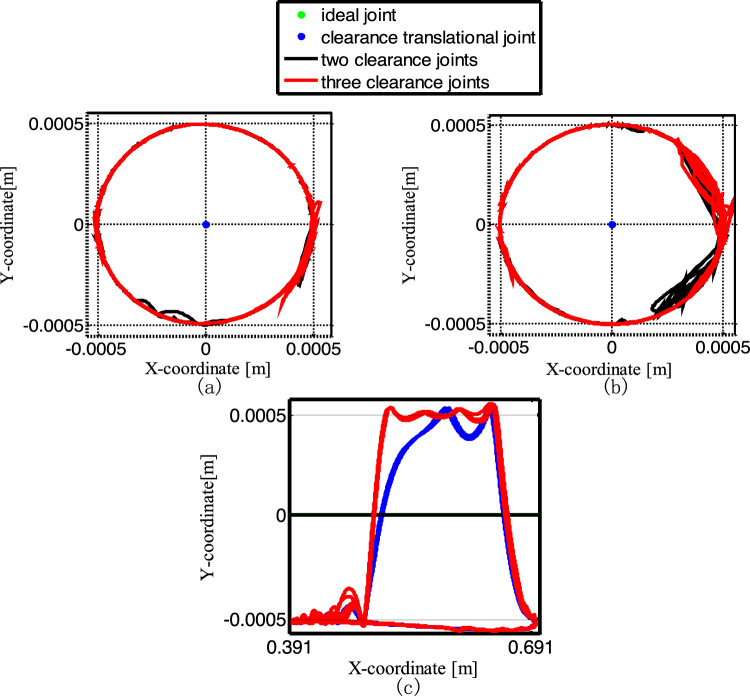
Center trajectory of the journal for different numbers of clearance joint: (**a**) the trajectory of clearance joints B; (**b**) the trajectory of clearance joints C; (**c**) the trajectory of clearance joints D.

Figure 26 shows the center trajectory of the journal in clearance joint under the four conditions. Figure 26a–c describe the trajectory of clearance joints B, C and D respectively. The data of the 5 cycles after the crank rotates 50 cycles are selected. As can be seen from Fig. 26, when the mechanism is ideal or have a clearance translational joint, the center trajectory of joint B and C is one point respectively.

In general, there are more collisions at joint C than at joint B, and the collisions mostly occur at the left and right ends of the journal, as shown by the red and black lines in Fig. 26a and b. The trajectory of the ideal mechanism at joint D is a line segment with x value from 0.391 m to 0.691 m. The trajectory of the mechanism with a clearance translational joint is shown in Fig. 26c, due to the influence of gravity, the slider mainly has contact and collision with the lower surface of the guide rail, and has two collisions with the upper surface. Compared with the mechanism with clearance translational joint, the contact period between the slider and the upper surface of the guide rail becomes larger in the mechanism with three clearance joints.

**Figure 27:**
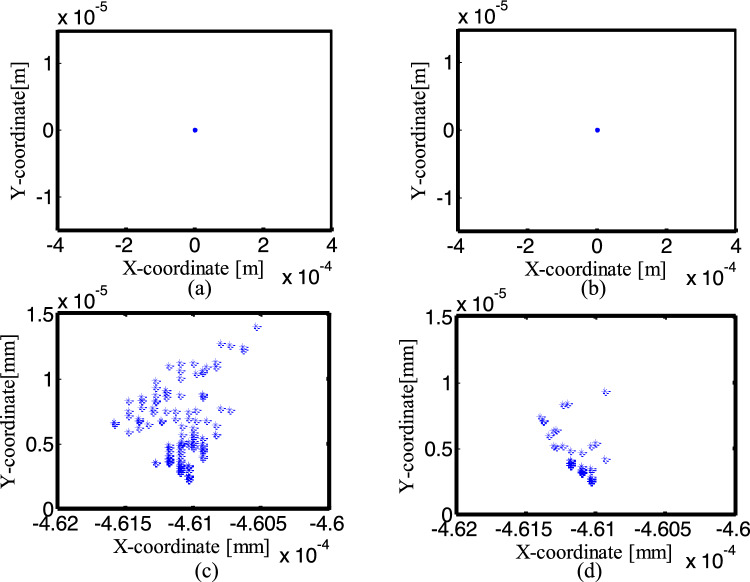
Poincaré maps of center trajectory at joint B for different number of clearance joint: (**a**) an ideal mechanism; (**b**) one clearance translational joint mechanism; (**c**) two revolute joints mechanism; (**d**) three clearance joints mechanism.

**Figure 28:**
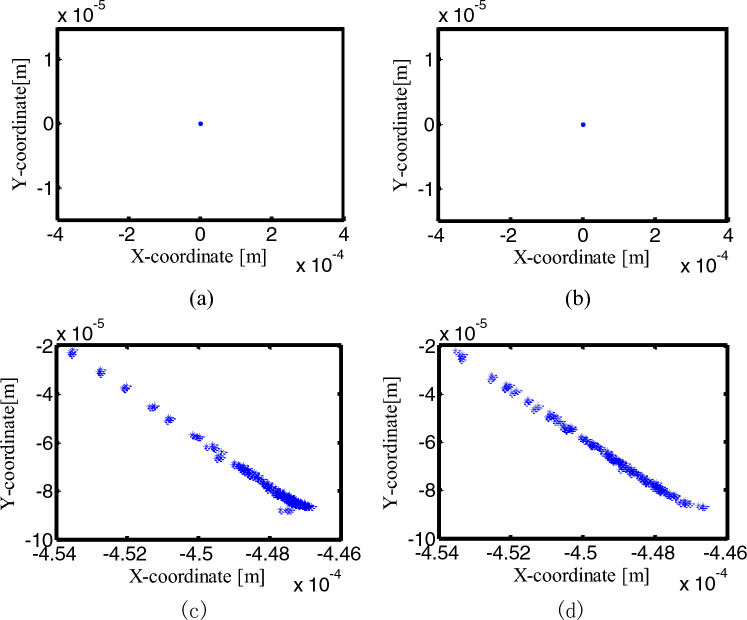
Poincaré maps of center trajectory at joint C for different number of clearance: (**a**) an ideal mechanism; (**b**) one clearance translational joint mechanism; (**c**) two revolute joints mechanism; (**d**) three clearance joints mechanism.

**Figure 29:**
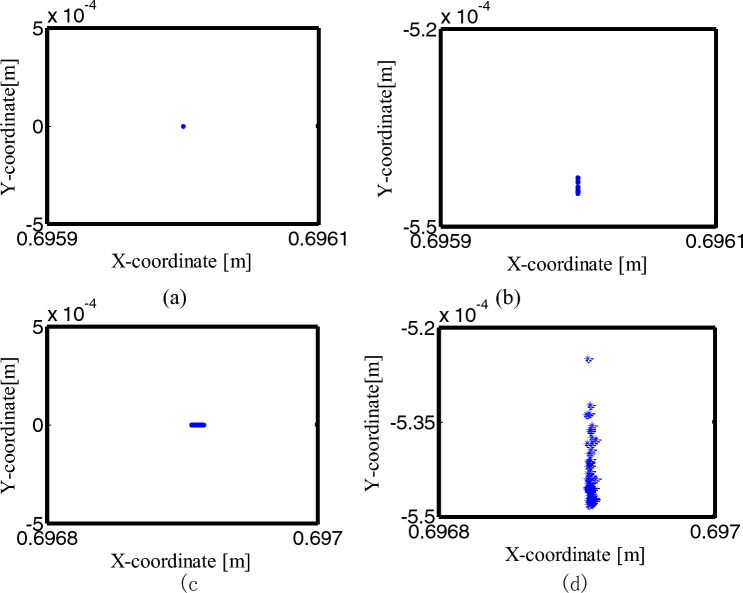
Poincaré maps of center trajectory at joint D for different number of clearance joint: (**a**) an ideal mechanism; (**b**) one clearance translational joint mechanism; (**c**) two revolute joints mechanism; (**d**) three clearance joints mechanism.

Figures 27, 28 and 29 are the Poincaré maps of the joints B, C and D under four conditions. Select 300 cycles after the crank rotates for 50 cycles, and take the x-direction and y-direction positions of the journal center when the crank angle is 0°. Figures 27, 28, 29a–d are Poincaré map for ideal mechanism, one clearance translational joint mechanism, two clearance revolute joints mechanism and three clearance joints mechanism.

From (a) to (b) in Figs. 27, 28, the Poincaré map of the ideal mechanism and the mechanism with a clearance translation joint at the joints B and C is a mapping point, because the joints B and C are ideal under the two conditions. For the mechanism with two clearance joints and the mechanism with three clearance joints, when the crank angle is 0°, the Poincaré mapping points of the center trajectory in joint B are mainly distributed in the region of the minimum value in the x-direction (close to − 0.0005). The mapping point of joint C is similar to joint B, and the x-direction is close to − 0.0005, see (c) and (d) in Figs. 27, 28.

In Fig. 29, the Poincaré mapping point of the ideal mechanism at joint D is a point, which means a periodic motion, the mapping point of a clearance translational mechanism at joint D is a constant value in the x-direction, the y-direction values are a relatively concentrated discrete point, and the motion in this state is quasi-periodic. Since the translational joint is ideal in the mechanism with two clearance revolute joints, the y value of the mapping point at joint D is constant, and the x value is relatively concentrated discrete point. In the mechanism with three clearance joints, the x value of the mapping point at the joint D is a small range near 0.6969 m, and the y value is in the range of − 0.00055 ~ − 0.00052 m, similar to the mechanism with two clearance revolute joints in the x-direction, and the mechanism with a clearance translational joint in the y-direction, the three-clearance joint mechanism has the characteristics of the two-clearance revolute joint mechanism and the one-clearance translational joint mechanism, it is not a simple superposition, but a nonlinear coupling of these multiple clearance joints.

**Figure 30:**
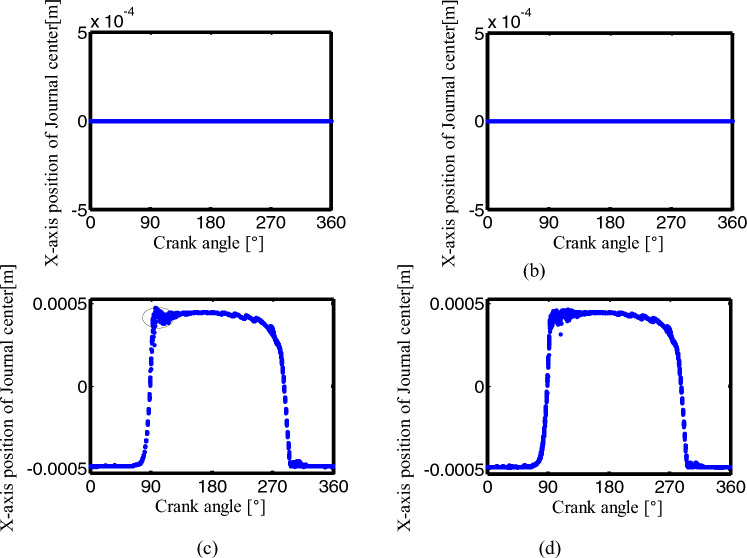
The center trajectory of the joint B in x-direction for different number of clearance joint: (**a**) an ideal mechanism; (**b**) one clearance translational joint mechanism; (**c**) two revolute joints mechanism; (**d**) three clearance joints mechanism.

**Figure 31:**
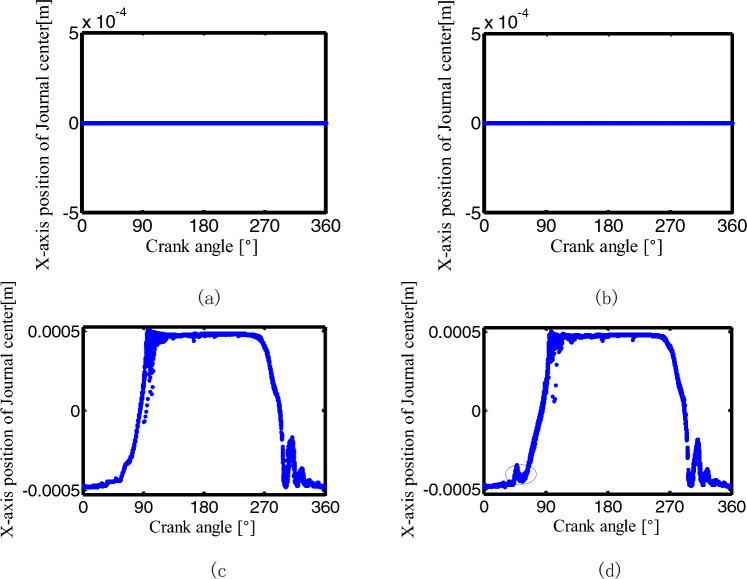
The center trajectory of the joint C in x-direction for different number of clearance joint: (**a**) an ideal mechanism; (**b**) one clearance translational joint mechanism; (**c**) two revolute joints mechanism; (**d**) three clearance joints mechanism.

Figures 30, 31 show the center trajectory of the joints B and C in the x-direction with the crank angle variation for the mechanisms with a different number of clearance joints. The data of 30 rotation cycles after the crank rotates for 50 cycles were selected.

**Figure 32:**
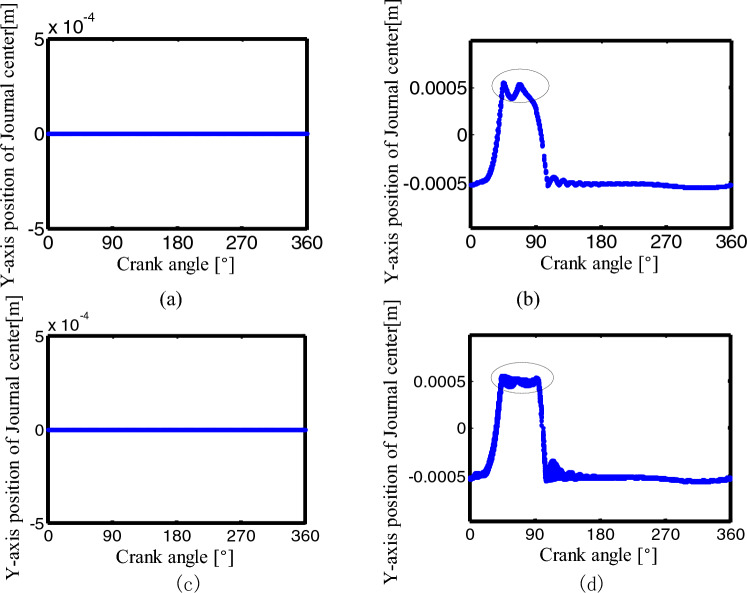
The center trajectory of the joint D in y-direction for different number of clearance joint: (**a**) an ideal mechanism; (**b**) one clearance translational joint mechanism; (**c**) two revolute joints mechanism; (**d**) three clearance joints mechanism.

The x-direction position of the journal center at joints B and C is constant in the first two conditions, see (a) and (b) in Figs. 30, 31. Mechanisms with two clearance joints and three clearance joints have similar graphics at joints B and C, mainly distribute in the maximum and the minimum value area, see Figs. 30, 31c,d, when the crank angle is in the range of $90^\circ -120^\circ$, the journal center trajectory of joints B and C in the x-direction is different, Relatively large peak appears in the former mechanism (see the small circle in Fig. 30c), while the trajectory in the latter mechanism is smoother(see Fig. 30d). The center trajectory of joint C appears a peak around 60° of the crank angle (see Fig. 31d at the small circle), this is caused by the collision of the clearance translational joint D(see the small circle in Fig. 32d), that is, the clearance joint D has a significant effect on the adjacent joint C.

Figure 32 shows the center trajectory of the joint D in y-direction under four different conditions. The data of 30 rotation cycles after the crank rotates for 50 cycles were selected.

In the ideal mechanism and the two-clearance joints mechanism, the joint D is ideal, so the y-direction displacement of the slider center is 0. When the mechanism has one clearance translational joint or three clearance joints, the y-direction displacement of the slider center at joint D is similar. The slider is in contact or collision with the upper surface when the crank angle is $30^\circ \sim 90^\circ$, at other angles, the slider is in contact or collision with the lower surface, see (b) and (d) in Fig. 32. Compared with the one-clearance translational mechanism, the three-clearance joints mechanism has two more clearance joints, which has a significant impact on the translational joint.

In general, as the number of clearance revolute joint increases, the dynamic response has a higher peak value and appears obvious aperiodic behavior. Compared to the clearance translational joint with the clearance rotate joint, the former has much less influence on the slider displacement, velocity, acceleration and joint force, the latter plays a leading role in the mechanism with the mixed clearance joints.

## Conclusion

Based on the principles of energy balance and momentum conservation, the dissipated energy calculation in the collision process was realized, and a modified contact force model that is not limited by the size of the coefficient of restitution was derived. The model can be used to calculate the contact force for perfectly elastic contact, inelastic contact and fully inelastic contact. The modified contact force model and the LuGre friction model were used as the normal force and tangential force in the dynamic contact process of the clearance joint. Combined with the Lagrangian equation of the first kind with the modified contact force model, the LuGre friction model, and the Baumgarte stabilization method, the dynamic equation of the multi-body system with clearance joints was established.

Simulation calculation for the mechanism under four conditions, namely the ideal mechanism, the mechanism with one clearance translational joint, the mechanism with two clearance revolute joints and the mechanism with three clearance joints (two clearance revolute joints and one clearance translation joint). Analyze the effects of different types of clearance joints and different numbers of clearance joints on multibody mechanisms. Numerical research showed that the peak frequencies of acceleration, joint force and crank moment increase after introducing the clearance translational joint. Compared to the clearance translational joint with the clearance revolute joint, the former has much smaller effect on the slider displacement, velocity, acceleration and joint force, which means that the clearance revolute joint plays a leading role in the dynamic response of the multi-clearance joint mechanism. Through the analysis of the slider displacement, velocity, acceleration, joint force and center trajectory Poincaré mapping, the dynamic response of the mechanism with multiple clearance joints is not a simple superposition of a single clearance joint, but the complex nonlinear relationship of these clearance joints. From the center trajectory of the journal, the trajectory is mainly distributed in the x-direction maximum and minimum values, which can predict the journal and bearing wear.

The dynamic modeling and analysis of the mechanism with clearance joints were discussed, but the problems of wear, lubrication, spatial joints, higher frequency experiments, computational efficiency and mechanism optimization were not considered in this paper. In the future studies, this type of issue will be investigated in detail.

## Data Availability

The data used to support the findings of this study are available from the corresponding author upon request.
